# *Arabidopsis* AtRRP44A Is the Functional Homolog of Rrp44/Dis3, an Exosome Component, Is Essential for Viability and Is Required for RNA Processing and Degradation

**DOI:** 10.1371/journal.pone.0079219

**Published:** 2013-11-07

**Authors:** Naoyoshi Kumakura, Hiroka Otsuki, Masayuki Tsuzuki, Atsushi Takeda, Yuichiro Watanabe

**Affiliations:** Department of Life Sciences, Graduate School of Arts and Sciences, the University of Tokyo, Tokyo, Japan; Université Libre de Bruxelles. BELGIQUE, Belgium

## Abstract

The RNA exosome is a multi-subunit complex that is responsible for 3ʹ to 5ʹ degradation and processing of cellular RNA. Rrp44/Dis3 is the catalytic center of the exosome in yeast and humans. However, the role of Rrp44/Dis3 homologs in plants is still unidentified. Here, we show that *Arabidopsis* AtRRP44A is the functional homolog of Rrp44/Dis3, is essential for plant viability and is required for RNA processing and degradation. We characterized AtRRP44A and AtRRP44B/SOV, two predicted *Arabidopsis* Rrp44/Dis3 homologs. AtRRP44A could functionally replace *S. cerevisiae* Rrp44/Dis3, but AtRRP44B/SOV could not. *rrp44a* knock-down mutants showed typical phenotypes of exosome function deficiency, 5.8S rRNA 3ʹ extension and rRNA maturation by-product over-accumulation, but *rrp44b* mutants did not. Conversely, AtRRP44B/SOV mutants showed elevated levels of a selected mRNA, on which *rrp44a* did not have detectable effects. Although T-DNA insertion mutants of AtRRP44B/SOV had no obvious phenotype, those of AtRRP44A showed defects in female gametophyte development and early embryogenesis. These results indicate that AtRRP44A and AtRRP44B/SOV have independent roles for RNA turnover in plants.

## Introduction

Eukaryotic 3ʹ to 5ʹ exoribonucleolytic activities are important for a wide range of reactions of RNA metabolism and maturation, including processing of small nuclear RNA (snRNA), small nucleolar RNA (snoRNA) and ribosomal RNA (rRNA), and mRNA decay. The main pathway of 3ʹ to 5ʹ exoribonucleolytic activity is the RNA exosome [1].

The exosome is evolutionarily conserved among eukaryotes. The exosome targets a vast range of RNAs at many stages of their lives, from maturation through constant quality control to final turnover. The exosome of eukaryotes is composed of eleven polypeptide components that can be divided into two major groups according to their structure and function [2-4].

The first group of exosome components comprises nine proteins that form the ‘exosome core’. Six of the subunits, Rrp41, Rrp42, Rrp45, Rrp43, Rrp46 and Mtr3 (each containing an RNase PH domain), are organized into a hexamer, capped on one side by a trimer of subunits Rrp40, Rrp4 and Csl4 (each containing an S1 RNA binding domain, and two of which contain a KH domain). In yeast, the loss of any one subunit of the exosome core is lethal, showing similar rRNA processing defect profiles [5,6]. Moreover, x-ray crystallographic analysis of the human exosome core revealed that all of the nine subunits are required for its structural integrity [7].

The exosome core itself has no catalytic activity in yeast and humans [7,8]. The active sites of the exosome are endowed by the second group components, Rrp6 and Rrp44/Dis3. Both Rrp6 and Rrp44/Dis3 have exoribonucleolytic activities. It was reported that Rrp6 resides in the nucleus of yeast cells, whereas in human cells Rrp6 is found in both the nucleus and cytoplasm [9]. Rrp44/Dis3 has both exoribonucleolytic and endoribonucleolytic activities and is essential for the activity of the yeast exosome. In yeast, the loss of Rrp44/Dis3 is lethal, the same as the loss of individual subunits of the exosome core.

In *Arabidopsis thaliana*, using tandem affinity purification of tagged AtRRP4 and AtRRP41, Chekanova et al. [10,11] characterized the exosome core containing nine conserved components. These components are essential for plant viability (except for CSL4) and target a wide range of RNAs including a selective subset of mRNAs, miRNA processing intermediates and noncoding RNAs [10]. It was reported that the exosome core, represented by AtRRP4 and AtRRP41, is also involved in siRNA-independent silencing of heterochromatic loci [12]. *Arabidopsis* also has the second group of exosome components, Rrp6 and Rrp44/Dis3. Lange et al. characterized three *Arabidopsis* Rrp6 homologs, AtRRP6L1, AtRRP6L2 and AtRRP6L3 [13]. AtRRP6L1 and -2 are targeted to the nucleus, whereas AtRRP6L3 is restricted to the cytoplasm. While AtRRP6L1 lacks the N-terminal PMC2NT domain that human and yeast Rrp6s have, AtRRP6L1 can complement the growth defect of the yeast *rrp6Δ* strain. AtRRP6L2 is involved in the degradation of rRNA maturation by-products, the accumulation of which is a typical phenotype of exosome defects [13]. However, *Arabidopsis* homologs of Rrp44/Dis3, the catalytic center of the exosome in yeast, have not been characterized in detail. From the *Arabidopsis* genome sequence, two Rrp44/Dis3 candidates are predicted, AtRRP44A and AtRRP44B/SOV (Suppressor Of Varicose) [10,14]. In a previous report, AtRRP44B/SOV was shown to localize in the cytoplasm and was suggested to be involved in mRNA decay [14]. In addition, T-DNA insertion mutants of AtRRP44A, the closest homolog of Rrp44/Dis3, showed a lethal phenotype. At present, whether AtRRP44A and/or AtRRP44B/SOV are *Arabidopsis* exosome components is an open question.

In this work, we characterized AtRRP44A and AtRRP44B/SOV. Yeast complementation assays revealed that AtRRP44A, but not AtRRP44B/SOV, could complement a *S. cerevisiae* Rrp44/Dis3 mutant. Analysis of the corresponding mutants and its target RNAs showed that AtRRP44A and the exosome core components AtRRP4 and AtRRP41 shared common features. In contrast, AtRRP44B/SOV showed a different target RNA profile and phenotype compared with AtRRP44A and the exosome core components. These results suggest that AtRRP44A is an *Arabidopsis* exosome component, like Rrp44/Dis3, but AtRRP44B/SOV is not.

## Results

### 1: AtRRP44A and AtRPP44B/SOV are *Arabidopsis* Rrp44/Dis3 candidates

*Arabidopsis* has two Rrp44/Dis3 candidates, previously designated AtRRP44A (AT2G17510) and AtRRP44B/SOV (AT1G77680) [10,14,15]. No other candidates with similarity were identified by phylogenetic tree analysis in previous reports or our search (data not shown).

**Figure 1:**
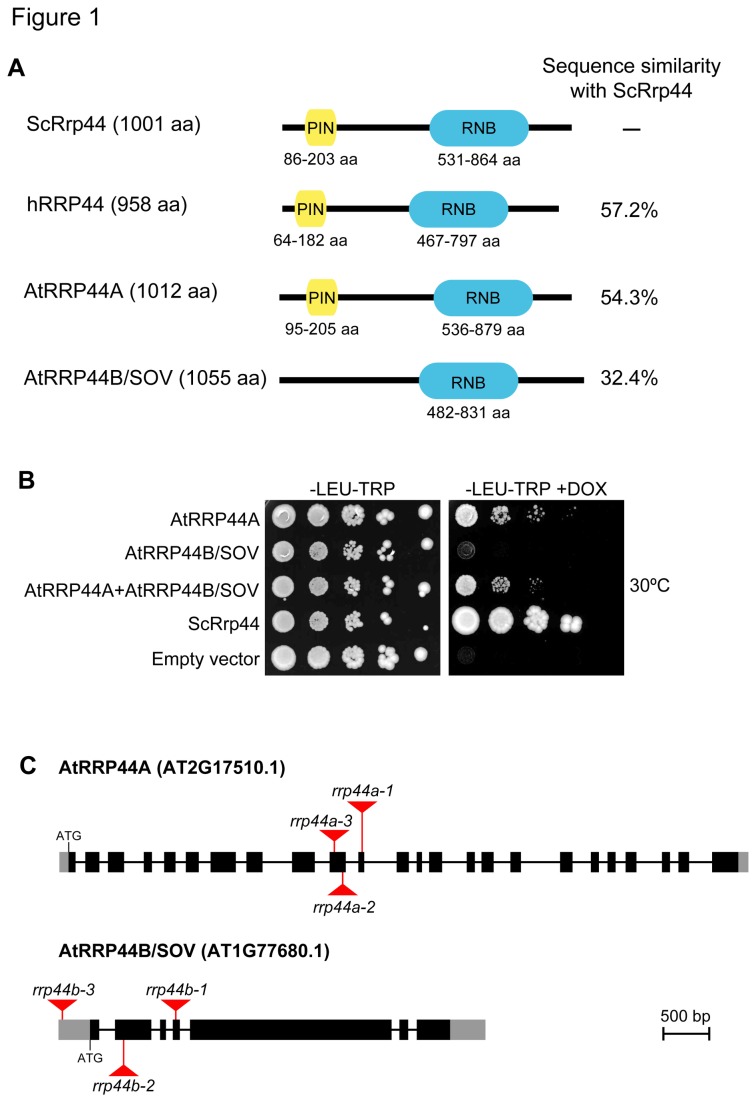
*Arabidopsis* Rrp44/Dis3 homologs. (A) Schematics of the Rrp44/Dis3 homologs *S. cerevisiae* Rrp44 (ScRrp44), human RRP44/DIS3 (hRRP44), and *A. thaliana* AtRRP44A and AtRRP44B/SOV. Yellow and blue boxes represent the PIN and RNB domains, respectively, that are conserved among Rrp44/Dis3 homologs. aa represents amino acids. (B) AtRRP44A complements the S. *cerevisiae* *rrp44* doxycycline (DOX) repressible mutant. Growth phenotypes resulting from the expression of plasmid-borne AtRRP44A, AtRRP44B/SOV, AtRRP44A and AtRRP44B/SOV, and ScRrp44 in *S. cerevisiae* BSY1883 strain, and negative control alleles were assessed in the presence (repressed chromosomal ScRrp44) or absence (expressed chromosomal ScRrp44) of DOX after incubation for 90 h at 30°C. –LEU-TRP, without leucine and tryptophan. (C) Diagram of the intron–exon structure of AtRRP44A and AtRRP44B/SOV. UTRs are indicated by grey boxes, exons by black boxes and introns by solid lines. T-DNA insertion sites for *rrp44a-1* (SALK_037533), *rrp44a-2* (SALK_141741), *rrp44a-3* (SALK_051800), *rrp44b-1* (SAIL_804_F05), *rrp44b-2* (SALK_017934) and *rrp44b-3* (SALK_010765) are shown in red arrowheads.

The common domains found among the *Arabidopsis* homologs, budding yeast and human Rrp44/Dis3s, are depicted in Figure 1A. Rrp44 of *Saccharomyces cerevisiae* (ScRrp44) contains two essential domains; the pilT N-terminal (PIN) and RNA binding (RNB) domains. It was reported that the PIN domain is crucial for Rrp44/Dis3 binding to the exosome core in humans and *S. cerevisiae* [16-18]. The RNB domain is responsible for exoribonuclease activity of Rrp44/Dis3 in humans, *S. cerevisiae* and *Schizosaccharomyces pombe* homologs [7,19,20]. AtRRP44A has both the PIN and RNB domains. Although AtRRP44B/SOV lacks the PIN domain, it has a highly-conserved RNB domain. Based on these highly conserved domains, we tested both AtRRP44A and AtRRP44B/SOV as functional homologs of *S. cerevisiae* Rrp44/Dis3. 

### 2: *Arabidopsis* AtRRP44A complements the growth defect of a *S. cerevisiae rrp44* knock-down mutant

Because the deduced amino acid sequences of AtRRP44A and AtRRP44B/SOV included essential domains of Rrp44/Dis3, we suspected that AtRRP44A and/or AtRRP44B/SOV would substitute for ScRrp44 function *in vivo*. To test whether the *Arabidopsis* AtRRP44A and/or AtRRP44B/SOV proteins could function in *S. cerevisiae*, we expressed the AtRRP44A and AtRRP44B/SOV proteins in *S. cerevisiae* strain BSY1883 [21]. In this strain, the essential chromosomal ScRrp44 copy is under the control of a doxycycline-repressible promoter. Thus, we could test the functional interchangeability between homologs in the presence of doxycycline (DOX) (Figure 1B). AtRRP44A could restore the growth defect of the DOX-repressible *scrrp44* strain. In contrast, AtRRP44B/SOV transformed *scrrp44* strain did not survive. The levels of complementation by AtRRP44A alone and together with AtRRP44B/SOV were almost the same, suggesting that AtRRP44A could replace ScRrp44. 

We also tested whether C-terminally hemagglutinin (HA)-tagged AtRRP44A protein (AtRRP44AHA) complemented the growth defect of the strain. As shown in Figure S1, AtRRP44AHA could not replace the function of ScRrp44 at certain temperatures. This data suggested that the intactness of the C-terminal structure and/or sequence of AtRRP44A were important *in vivo*.

### 3: Establishment of tissue-specific knock-down mutants of AtRRP44A

Next, we intended to test whether AtRRP44A and/or AtRRP44B/SOV were actually involved in RNA turnover. We first collected several T-DNA tagged mutants from seed stock centers. We obtained some lines in which the AtRRP44A gene was disrupted with T-DNA (*rrp44a-1*, *rrp44a-2* and *rrp44a-3* in Figure 1C) as heterozygotes but failed to obtain homozygous segregants among their siblings (Table S1), as previously reported [14]. This implied that the AtRRP44A gene is essential for plant viability and that plants could not grow in the absence of AtRRP44A gene function. However, we did obtain homozygous T-DNA insertion segregants from T-DNA tagged AtRRP44B/SOV lines (*rrp44b-1*, *rrp44b-2* and *rrp44b-3* in Figure 1C). This implied that the function of AtRRP44B/SOV is not essential for plant viability.

**Figure 2:**
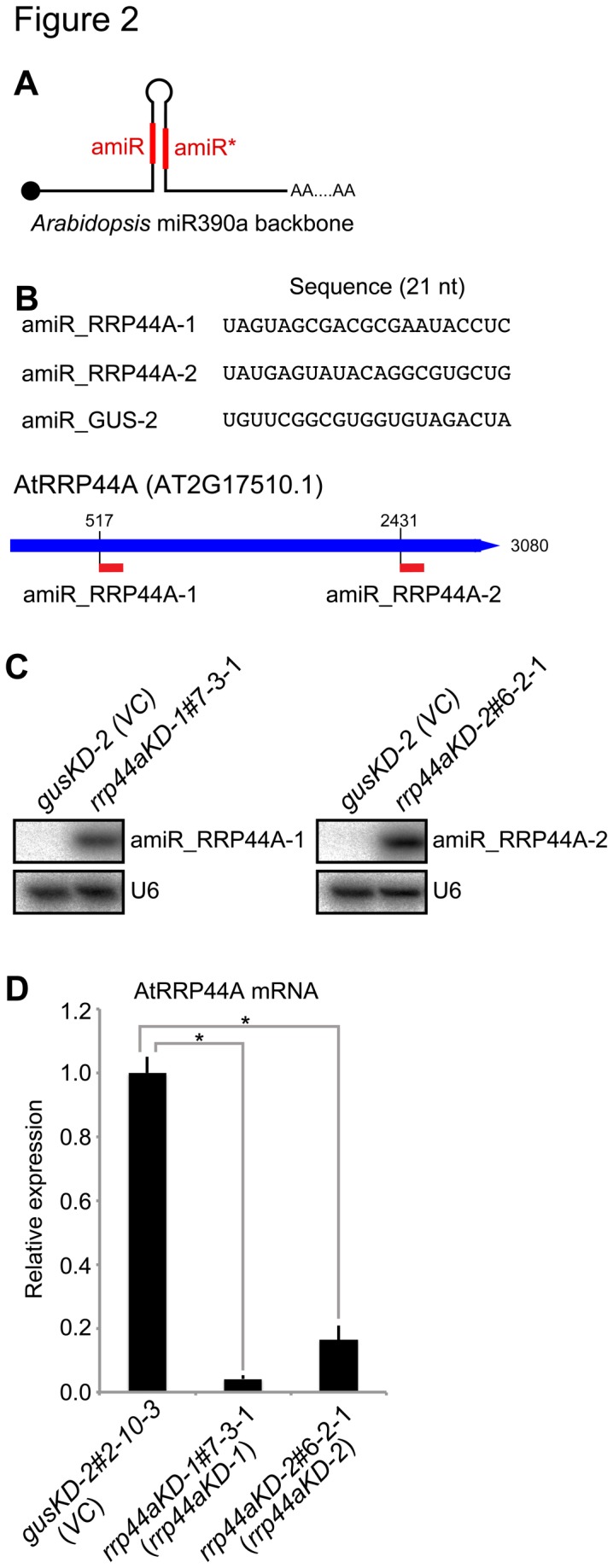
Establishment of *rrp44a* knock-down mutants by artificial microRNA (amiR). (A) Schematics of the amiR precursor. A black circle represents cap structure. amiR and amiR* represent guide strand and passenger strand, respectively. amiR sequences targeting AtRRP44A (amiR_AtRRP44A-1 and amiR_AtRRP44A-2) and *E. coli* β-glucuronidase (GUS) (amiR_GUS-2; vector control with no target sites in the *Arabidopsis* genome) are shown. (B) AmiR sequences and target sites in the AtRRP44A (AT2G17510) mRNA. (C) Expression of amiR_RRP44A-1 and amiR_RRP44A-2 were detected by small RNA gel blot analysis. Small RNA gel blots were hybridized with an antisense oligonucleotide complementary to the amiRs. U6 RNA (U6) served as a loading control for small RNA. Total RNAs were isolated from 25 days post-germination (dpg) rosette leaves of T3 homozygous lines carrying a unique insertion in Col-0 background plants expressing amiR_RRP44A-1 (AtRRP44A Knocked Down-1; *rrp44aKD-1#*7-3-1), amiR_RRP44A-2 (*rrp44aKD-2*#6-2-1), or amiR_GUS-2 (gusKD-2#2-10-3: vector control (VC)). (D) The amounts of AtRRP44A mRNA in *gusKD-2*#2-10-3, *rrp44aKD-1*#7-3-1 and *rrp44aKD-2*#6-2-1 were analyzed by qRT-PCR. Total RNAs were isolated from 25 dpg rosette leaves. Error bars represent standard errors. Six biological replicates and two technical replicates were performed. * indicates significant difference (*p* < 0.01, Tukey’s test) between *gusKD-2* (VC) and *rrp44aKD-1* and *-2*.

As mentioned above, we could not obtain homozygous AtRRP44A mutants. Therefore, we introduced two strategies to study the RNA profiles in conditions where AtRPP44A function was lowered, but not lost, throughout the life cycle. First, we tried to use an artificial microRNA (amiR) strategy [22,23] to knock down AtRRP44A expression and establish feasible mutants. The amiR strategy can downregulate target mRNAs efficiently and also avoid possible off-target effects. Two distinct 21-nt sequences, designated amiR_RRP44A-1 and amiR_RRP44A-2 (Figure 2B), both targeting different sites of the AtRRP44A mRNA, were designed using the WMD3 micro RNA designer program [23]. The amiR_GUS-2 sequence (Figure 2B) targeting the *E. coli* β-glucuronidase sequence was designed as a negative control miRNA (no candidate targets in the *A. thaliana* genome). The mature *Arabidopsis* miR390 sequence was replaced with each designed amiR sequence in the *Arabidopsis* pri-MIR390A (AT2G38325) sequence context (Figure 2A) [24]. Second, to avoid lethal phenotypes, we expressed these amiRs specifically in mesophyll cells under the chlorophyll a/b-binding protein 3 (CAB3) promoter [25,26]. CAB3 is well known as a gene specifically expressed in mesophyll cells. The activity of the CAB3 promoter was confirmed in mesophyll cells by promoter GUS assay (Figure S2). 

Wild-type Col-0 plants were floral-dipped and transformed with the amiRNA expression vectors, amiR_RRP44A-1, amiR_RRP44A-2 or amiR_GUS-2, separately. After self-crossing, homozygous lines carrying a single insertion were selected, designated *rrp44aKD-1#7-3-1*, *rrp44aKD-2#6-2-1* and *gusKD-2#2-10-3*, respectively. The expression of amiR_RRP44A-1 and amiR_RRP44A-2 was confirmed in *rrp44aKD-1#7-3-1* and *rrp44aKD-2#6-2-1*, respectively, by small RNA gel blot analysis (Figure 2C). 

Then, the levels of target AtRRP44A mRNA in leaves were assessed by quantitative real-time PCR (qRT-PCR). Levels of the mRNA decreased to 5% in *rrp44aKD-1*#7-3-1 and 16% in *rrp44aKD-2*#6-2-1 plants compared with *gusKD-2*#2-10-3 control plants (Figure 2D). These plants showed normal growth compared with Col-0 plants (data not shown). Thus, we successfully established two independent AtRRP44A knock-down lines, *rrp44aKD-1*#7-3-1 and *rrp44aKD-2*#6-2-1, and a vector control line, *gusKD-2*#2-10-3, hereafter designated the *rrp44aKD-1*, *rrp44aKD-2* and *gusKD-2* lines, respectively.

AtRRP4 and AtRRP41, the exosome core components, were also knocked down in the same way as AtRRP44A to compare their biological functions. As shown in Figure S3, AtRRP4 and AtRRP41 were successfully knocked down and two homozygous lines carrying a single insertion were selected for each, designated *rrp4KD-2* and *rrp4KD-3*, and *rrp41KD-1#3-3* (hereafter referred as *rrp41KD-1*) and *rrp41KD-1#9-2*, respectively.

### 4. AtRRP44A participates in 3ʹ processing of 5.8S rRNA, but AtRRP44B/SOV does not.

**Figure 3:**
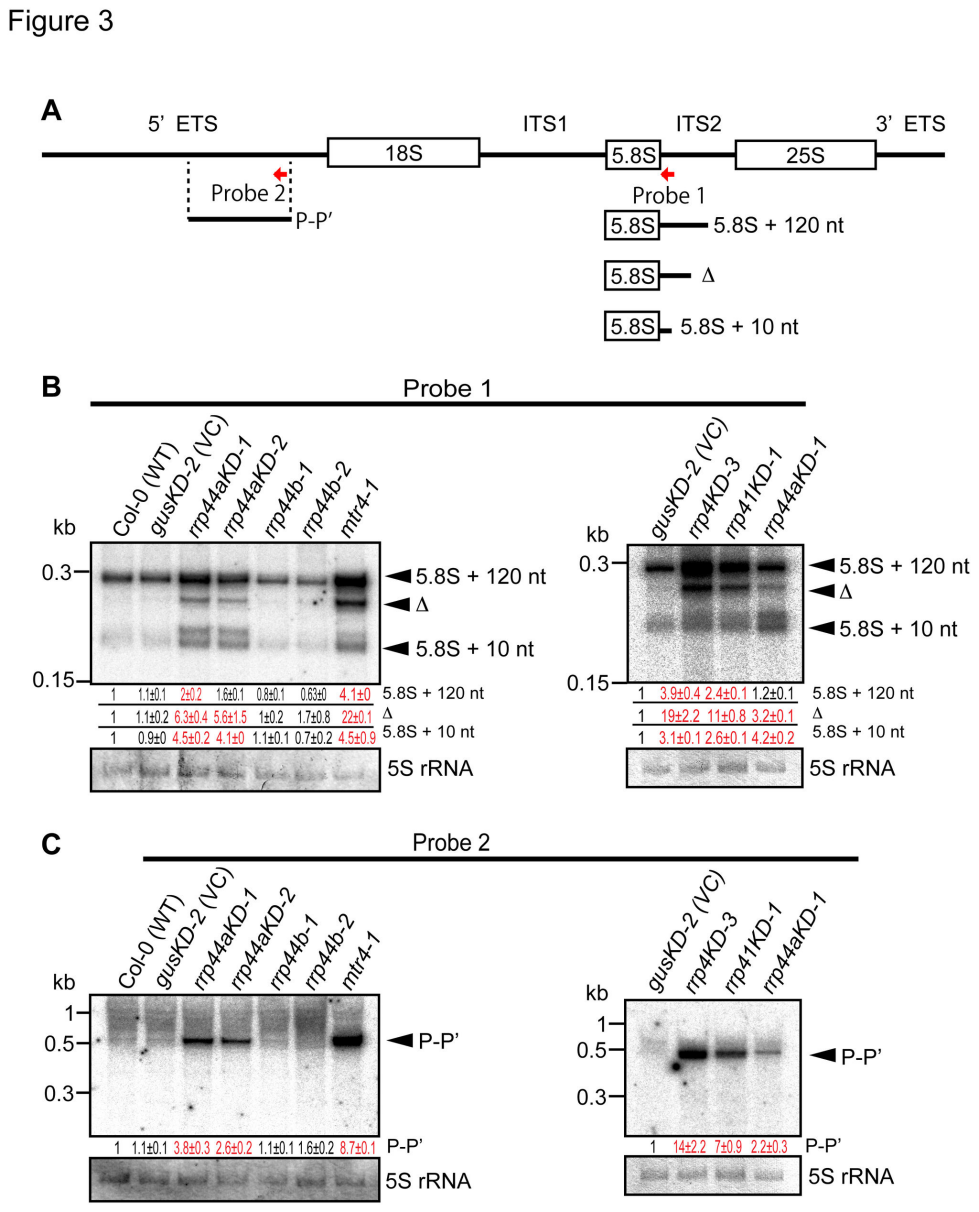
Analysis of rRNA processing and degradation. (A) Diagram illustrating the 5.8S rRNA processing intermediates and the rRNA maturation by-product generated from the 5ʹ ETS (P-Pʹ) compared with the 35S precursor [27]. Horizontal red arrows represent the positions of oligonucleotide probes used in this study. (B) The 5.8S rRNA 3ʹ extension is processed by AtRRP44A, AtRRP4 and AtRRP41, but not AtRRP44B/SOV. (C) The 5ʹ ETS is degraded by AtRRP44A, AtRRP4 and AtRRP41, but not AtRRP44B/SOV. RNA gel blots of 5.8S rRNA precursors (B) or the 5ʹ ETS (C). Total RNAs were isolated from 10 dpg rosette leaves of Col-0 (wild type: WT), *gusKD-2* (VC), *rrp44aKD-1*, *rrp44aKD-2*, *rrp44b-1*, *rrp44b-2* and *mtr4-1* plants or from *gusKD-2*, *rrp4KD-3*, *rrp41KD-1* and *rrp44aKD-1* plants (B and C). *mtr4-1* was used to determine the sequence of 5.8S processing intermediates [27]. Total RNAs were separated on 6% polyacrylamide gels. Methylene blue staining of 5S rRNA is shown as a loading control. Relative RNA levels estimated from band signals are indicated at the bottom of each lane as mean values ± SE with RNA levels in Col-0 plants set to 1.0.. Values for which P<0.05 (Tukey’s test) compared to corresponding wild type plants (*gusKD-2* or Col-0) were shown in red. Two (B and C: Left panels) or three (B and C: Right panels) biological replicates were performed for all RNA gel blots.

It was reported that accumulation of 5.8S rRNA 3ʹ-extended intermediates was increased by the depletion of yeast Rrp44/Dis3 or the exosome core components [5]. To test whether *Arabidopsis* AtRRP44A and/or AtRRP44B/SOV are similarly involved in 5.8S rRNA 3ʹ end processing, we performed northern blot analysis using a probe (probe 1, in Figure 3A) specific for the 3ʹ region downstream of the mature 5.8S rRNA sequence. *Arabidopsis* AtMTR4, a putative integral factor of the TRAMP complex, which stimulates exosome activity by addition of short poly (A) tails to its RNA substrates in yeast and human, is involved in 5.8S rRNA 3ʹ end processing [27]. In its absence, 5.8S rRNA intermediates with some extended sequence at the 3ʹ end would appear. Three 5.8S rRNA intermediates in *mtr4-1* (illustrated in Figure 3A) were characterized and assigned by Lange et al. [27]. When we analyzed our *rrp44aKD-1* and *rrp44aKD-2* lines, an increase of 5.8S rRNA processing intermediates with 3ʹ-end extensions was detected as in *mtr4-1*, but not in wild type plants (Figure 3B, left panel). Conversely, the 3ʹ end of the 5.8S rRNA was properly and efficiently processed in the *rrp44b-1* and *rrp44b-2* lines, which were indistinguishable from wild type plants (Figure 3B, left panel). These data suggested that AtRRP44A, but not AtRRP44B/SOV, is involved in 3ʹ end processing of 5.8S rRNA. 

It was reported that Rrp44/Dis3 interacts with the exosome core via the PIN domain [16-18] and is the essential catalytic component of the exosome core in humans and yeast [7,19]. In *Arabidopsis*, AtRRP4 and AtRRP41 are relatively well-characterized exosome core components [10,11]. To compare the roles of AtRRP44A and the exosome core components (represented by AtRRP4 and AtRRP41) in rRNA processing activities at the molecular level, we analyzed 5.8S rRNA processing intermediates in northern blot analysis and found similarly retarded 3ʹ end processing in the *rrp4KD-3*, *rrp41KD-1* and *rrp44aKD-1* lines (Figure 3B, right panel).

### 5: AtRRP44A participates in degradation of rRNA maturation by-products, but AtRRP44B/SOV does not

It was reported that the so-called 5ʹ external transcribed spacer (5ʹ ETS), known as a maturation by-product of rRNA synthesis, is a substrate of exosomes in yeast and plants [13,27,28]. Thus, we examined the possible involvement of AtRRP44A and AtRRP44B/SOV in degradation of the 5ʹ ETS. Known by-products, especially the P–Pʹ fragment, which was characterized as the substrate of AtMTR4 [27], were compared between the *rrp44aKD-1*, *rrp44aKD-2*, *rrp44b-1*, *rrp44b-2* and *mtr4-1* lines. A significant increase of P–Pʹ signals was detected in the *rrp44aKD-1*, *rrp44aKD-2* and *mtr4-1* lines compared with Col-0 and *gusKD-2*, but not in the *rrp44b-1* or *rrp44b-2* lines (Figure 3C, left panel). The accumulation of P–Pʹ fragments in the *rrp44aKD-1* and *rrp44aKD-2* lines indicated that AtRRP44A, but not AtRRP44B/SOV, was required for efficient degradation of rRNA maturation by-products. Together, our results suggested that AtRRP44A is required for 5.8S rRNA intermediate processing and the degradation of 5ʹ ETS rRNA maturation by-product (Figure 3B and C), similarly to ScRRP44 [17,29]. 

Total RNAs from *rrp4KD-3* and *rrp41KD-1* plants were also analyzed by northern blotting. As shown in the right panel of Figure 3C, 5ʹ ETS P–Pʹ fragments were significantly increased in the *rrp44aKD-1*, *rrp4KD-3* and *rrp41KD-1* lines (about 2, 14, and 7 times), compared with *gusKD-2* plants. Together, these results supported that AtRRP44A contributes to the same ribosomal processing pathway as other previously characterized exosome core components, AtRRP4 and AtRRP41.

### 6: AtRRP44A and AtRRP44B/SOV have different target RNA profiles

**Figure 4:**
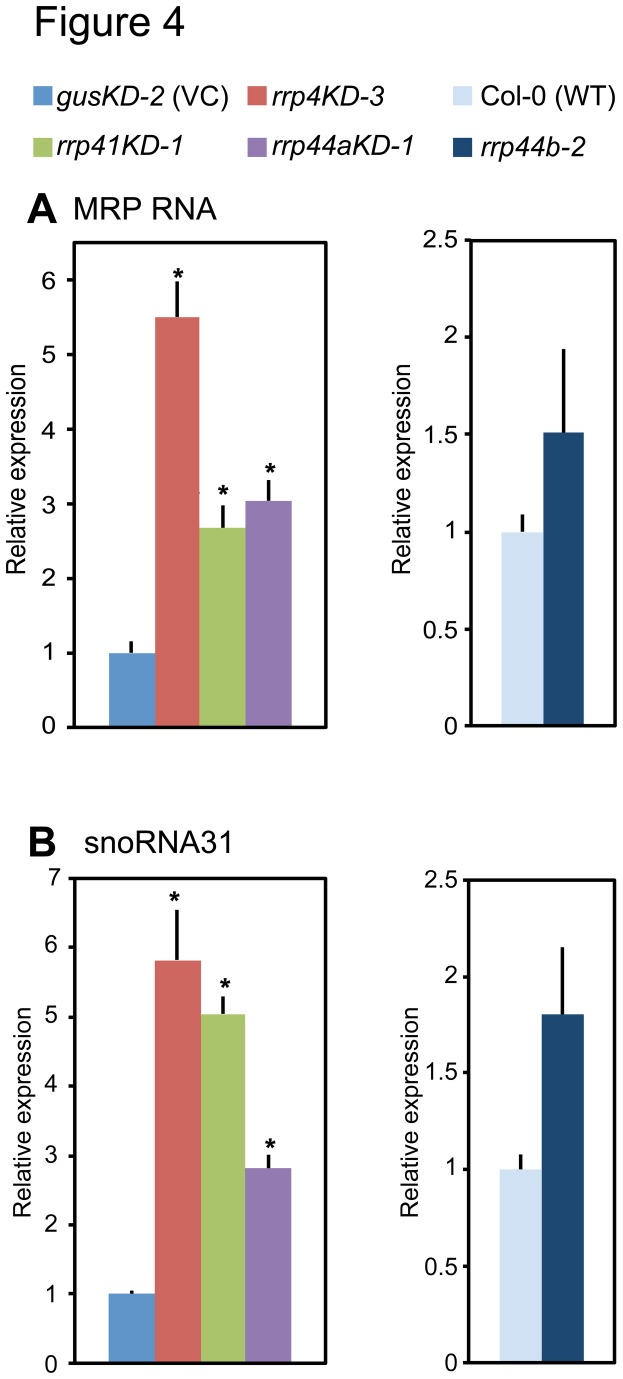
Levels of MRP RNA and snoRNA31 in *rrp44aKD-1*, *rrp44b-2* and the exosome core mutants. (A and B) qRT-PCR revealed that accumulation of the MRP RNA and snoRNA31 was upregulated in *rrp4KD-3*, *rrp41KD-1* and *rrp44aKD-1*, but not in *rrp44b-1*. AtRRP4 and AtRRP41 represent the *Arabidopsis* exosome core. Total RNAs were isolated from 10 dpg rosette leaves. EF1a mRNA was used as an endogenous control. Error bars represent standard errors. Three biological replicates and two technical replicates were performed. * indicates significant difference (*p* < 0.05, Tukey’s test) between mutant and wild type plants.

AtRRP44A, AtRRP4 and AtRRP41 were shown to be involved in rRNA processing, but AtRRP44B/SOV was not. Chekanova et al. (2007) discovered that the accumulation of subsets of RNAs was elevated in *rrp4* ^*iRNAi*^ and *rrp41* ^*iRNAi*^ lines (estradiol-inducible RNA interference lines of AtRRP4 and AtRRP41, respectively) using tiling arrays [10]. To investigate whether knock down or knock out of *AtRRP44A* or *AtRRP44B/SOV* affected exosome target RNAs, we chose MRP RNA and snoRNA31, which are required for rRNA processing and were found to be increased in *rrp4* ^*iRNAi*^ and *rrp41* ^*iRNAi*^ **[10], and examined their transcript levels in *rrp4KD-3*, *rrp41KD-1*, *rrp44aKD-1* and *rrp44b-2* using qRT-PCR (Figure 4). The levels of both RNAs increased in *rrp4KD-3*, *rrp41KD-1* and *rrp44aKD-1*, but not in *rrp44b-1*. This indicated that AtRRP44A, much like AtRRP4 and AtRRP41, is possibly required for proper control of MRP RNA and snoRNA31 accumulation.

**Figure 5:**
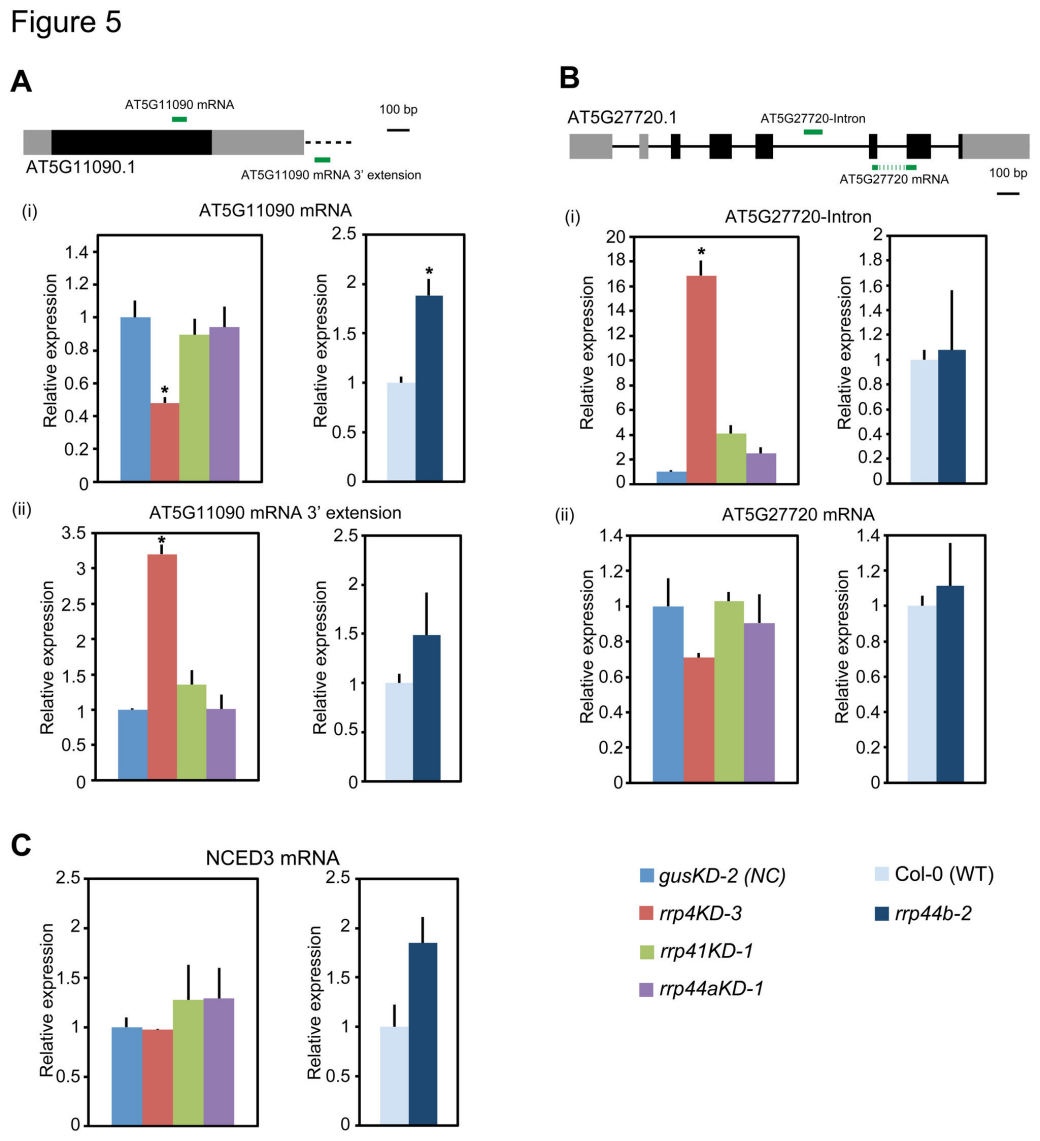
Levels of selected subsets of RNAs in *rrp44aKD-1*, *rrp44b-2* and the exosome core mutants. qRT-PCR analysis of total RNAs isolated from 7 dpg leaves for AtRRP4 and AtRRP41 (AT5G11090 3ʹ extension, AT5G27720-Intron) and AtRRP41L (NCED3) substrates [10,30] (A–C). UTRs are indicated by grey boxes, exons by black boxes, introns by solid lines and the 3ʹ extended region by a black broken line (A and B). Green lines show the coverage of amplicons used for qRT-PCR. Error bars represent standard errors. Three biological replicates and two technical replicates were performed. EF1a mRNA was used as an endogenous control. * indicates significant difference (*p* < 0.05, Tukey’s test) between mutants and *gusKD-2* (VC) or Col-0 (WT) plants.

Chekanova et al. also reported a number of mRNAs with extended 3ʹ ends [10]. It is thought that aberrantly lengthened mRNAs are degraded by exosomes. If AtRPP44A or AtRRP44B/SOV is responsible for exosome activity, the respective knock-down or knock-out lines should show an increase of lengthened mRNAs. Thus, we checked for accumulation of such mRNAs in the *rrp44aKD* and *rrp44b* mutant lines along with other characterized exosome core mutant lines. As shown in Figure 5A (ii), *AT5G11090* mRNA 3ʹ end extension was not observed in any of the mutant lines except for *rrp4KD*. However, the level of *AT5G11090* mRNA was elevated only in the *rrp44b* lines (Figure 5A (i)). These data indicated that AtRRP44B/SOV targets *AT5G11090* mRNA, but AtRRP44A and the exosome core components do not.

It was found that some excised introns and/or unspliced mRNAs accumulate in *rrp4* ^*iRNAi*^ [10]. Of all such RNAs reported previously, we checked the *AT5G27720* 5th intron as a representative in the *rrp44aKD* and *rrp44b* mutants. The results showed only slight increases in intron and absolute mRNA levels of *AT5G27720* compared with wild type plants (Figure 5B (i) and (ii)). 

Finally, we tested *NCED3* mRNA (Figure 5C). Yang et al. reported that *NCED3* mRNA accumulated in the T-DNA insertion *Arabidopsis* mutant of RRP41L, a component of exosome core [30]. The significant differences were not observed in the *NCED3* mRNA level in any of the mutants we used or wild type-plants. These data suggested that AtRRP44A, AtRRP44B/SOV and RRP41L have at least partially independent functions.

### 7: AtRRP44A, AtRRP44B/SOV and AtRRP41 do not affect Turnip crinkle virus genome RNA accumulation

We were interested in whether AtRRP44A, AtRRP44B/SOV and AtRRP41 could affect the accumulation of exogenous plant viral RNAs. In mammal cells, the exosome complex degrades Moloney murine leukemia virus (MLV) mRNA mediated by the zinc finger antiviral protein (ZAP), which binds to hRrp46, a component of the human exosome core [31]. These reports prompted us to check whether AtRRP44A, AtRRP44B/SOV and AtRRP41 affect plant viral RNA accumulation. We inoculated Turnip crinkle virus (TCV), a plant RNA virus with a single-stranded RNA genome [32] (Figure S4A) onto the *rrp44aKD*, *rrp44b and rrp41KD* lines. A purified virus preparation was inoculated at 16 days post-germination (dpg) onto the 4th and 5th rosette leaves, which were then harvested at 3 days post-inoculation (dpi). The accumulation of TCV genomic RNA was quantified by qRT-PCR (Figure S4 B–D). We could not detect any significant differences between wild type plants and the mutant lines. This implied that AtRRP44A, AtRRP44B/SOV and AtRRP41 did not impact on TCV RNA accumulation and are not involved in significant virus resistance.

### 8: AtRRP44A is required for female gametophyte development and early embryogenesis

**Figure 6:**
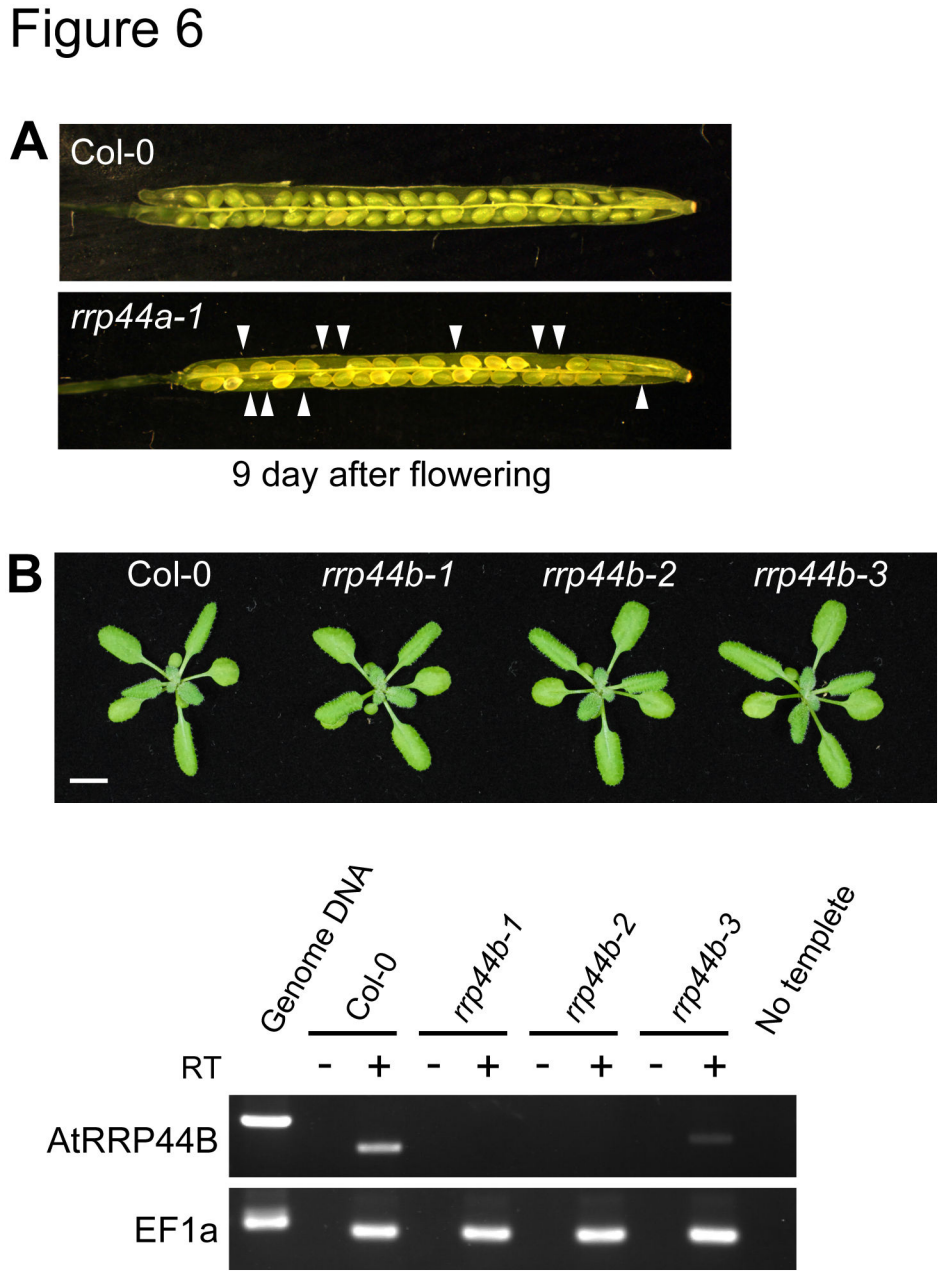
Characterization of AtRRP44A and AtRRP44B/SOV T-DNA insertion mutants. (A) Semi-sterility phenotype of the *rrp44a-1* mutant. (B) *rrp44b-1*, *rrp44b-2* and *rrp44b-3* have normal phenotypes. *rrp44b-1* and *rrp44b-2* mutants are RNA null. *rrp44b-3* mutants showed reduced RNA levels. Reverse Transcriptional PCR analysis of AtRRP44B/SOV, using total RNA isolated from 24 dpg *rrp44b-1*, *rrp44b-2* and *rrp44b-3* homozygous mutants. All samples showed similar levels of EF1a mRNA (Lower), but AtRRP44B/SOV mRNAs were not detectable (in *rrp44b-1* and *rrp44b-2*) or were decreased (in *rrp44b-3*). Primers used for this analysis are listed in Table S3.

It has been reported that AtRRP44A is required for normal female gametophyte development [14]. To investigate whether AtRRP44A is also required for male gametophyte development, we tested our mutant lines. We obtained T-DNA insertion mutants of AtRRP44A as shown in Figure 1C. As reported previously [14], siblings with homozygous insertions were not obtained (Table S1 and Figure 6A). The *rrp44a* heterozygotes germinated normally (Table S2). Though *rrp44a* mutant alleles were transmitted normally through the male parents, they were less frequently transmitted through the female (Table 1). This confirmed that AtRRP44A is required for female gametophyte development and early embryogenesis. 

**Table 1:** Reciprocal crosses between *rrp44a/*+ mutants and wild type (Col-0) plants.

Maternal parent of the cross	Paternal parent of the cross	*rrp44a/*+ progeny	wild type progeny	Number of F1 seeds examined
Col-0	*rrp44a-1/+*	48%	52%	160
Col-0	*rrp44a-2/+*	42%	58%	144
Col-0	*rrp44a-3/+*	53%	47%	94
*rrp44a-1/+*	Col-0	13%	87%	152
*rrp44a-2/+*	Col-0	9%	91%	134
*rrp44a-3/+*	Col-0	37%	63%	96

Zhang et al. reported that the *sov-1* mutant was indistinguishable from Col-0 plants [14]. AtRRP44B/SOV was reported to be involved in cytoplasmic mRNA decay. To confirm the phenotypes of *rrp44b*, three T-DNA insertional mutants were obtained. The T-DNA insertion sites are shown in Figure 1C. The additional two mutants were also indistinguishable from Col-0 plants (Figure 6B). *rrp44b-1* and *rrp44b-2* were null mutants; *rrp44b-3* showed much reduced AtRRP44B/SOV mRNA levels (Figure 6B).

## Discussion

In this study, we characterized *Arabidopsis* AtRRP44A and AtRRP44B/SOV as Rrp44/Dis3 homolog candidates. AtRRP44A complemented the growth defect of the *S. cerevisiae rrp44* inducible-repression mutant, but AtRRP44B/SOV did not (Figure 1B). We also showed that AtRRP44A and exosome core components target the same RNAs. The levels of 5.8S rRNA intermediates (Figure 3B), rRNA maturation by-products (Figure 3C), MRP RNA (Figure 4A) and snoRNA31 (Figure 4B) were all regulated in parallel by AtRRP44A and the exosome core components, AtRRP4 and AtRRP41. The molecular phenotypes of *rrp44aKD* mutants were weaker than that of *rrp4KD*, *rrp41KD* and *mtr4*. This implied that the contribution of AtRRP44A to rRNA processing was less than those of AtRRP4, AtRRP41 and AtMTR4. In contrast to AtRRP44A, AtRRP44B/SOV was not involved in the regulation of these RNAs (Figures 3 and 4). These data suggested that *Arabidopsis* AtRRP44A is a functional homolog of the yeast exosome component Rrp44/Dis3, but AtRRP44B /SOV is not.

The notion is supported by the same localization pattern of the exosome core components (AtRRP4, AtRRP41 and AtRRP45B) and AtRRP44A in previous reports [14,33,34]. In contrast, the *rrp44b* phenotype and the target RNAs of AtRRP44B/SOV were different from those of the exosome core mutants (Figures 3, 4, 5 and 6). In addition, AtRRP44B/SOV localizes to cytoplasmic foci [14]. These data suggest that AtRRP44B/SOV works independently from the exosome. Therefore, we think that it is suitable to call the AT1G77680 gene product SOV to avoid further confusion in the nomenclature of exosome components.

In yeast and human cells, the exosome core binds to Rrp44/Dis3 via the PIN domain. Although AtRRP44A was not immunoprecipitated with AtRRP4 or AtRRP41 [10], AtRRP44A has PIN domain. Therefore it is possible that AtRRP44A binds to the exosome core in the other experimental condition. Actually, our data indicate AtRRP44A and the exosome core function in the same pathway of RNA processing and degradation. 

**Figure 7:**
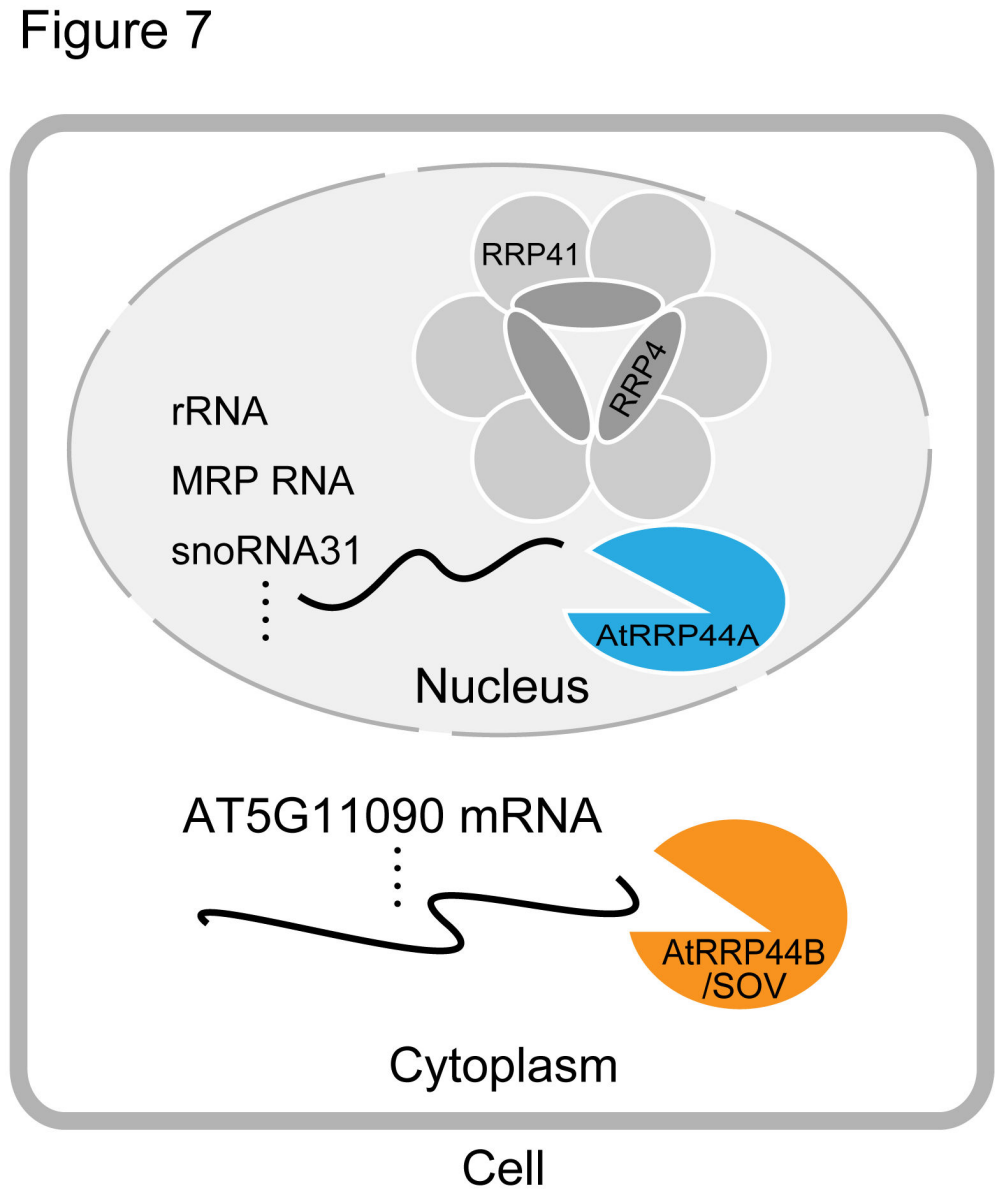
Model for the roles of *A. thaliana* AtRRP44A and AtRRP44B/SOV in RNA processing and degradation. AtRRP44A localizes to the nucleus and processes rRNAs with the exosome complex. However, AtRRP44B/SOV localizes to the cytoplasm and targets a select subset of mRNAs.

rRNA processing defects are common features of exosome core member mutants, and Rrp6 and Rrp44/Dis3 deficient mutants in *S. cerevisiae*. In *Arabidopsis*, the same molecular phenotype was observed in the putative exosome cofactor mutants *rrp6l2* [13] and *mtr4* [27] (a putative subunit of the TRAMP complex, which recruits RNAs to the exosome). AtRRP6L2 and AtMTR4 both localized to nuclei, suggesting that they form the nuclear exosome with the exosome core and AtRRP44A (Figure 7). 

In *rrp44b*, the level of AT5G11090 mRNA, one of three tested mRNAs, was elevated (Figure 5A(i)) being consistent with a previous report that suggested AtRRP44B/SOV selectively degrades certain subsets of mRNAs in the cytoplasm [14]. In human and fission yeast, DIS3L2, the closest homolog of AtRRP44B/SOV favorably degrade 3ʹ uridylated RNAs [35,36]. Future work testing whether AtRRP44B/SOV specifically degrades uridylated RNAs or not clarify this area. 

Zhang et al. reported that AtRRP44B/SOV localized to cytoplasmic foci. In *S. pombe*, DIS3L2, the closest homolog of AtRRP44B/SOV, also forms cytoplasmic foci that partially co-localize with P-bodies, which are well characterized cytoplasmic foci required for mRNA degradation and storage [37,38]. Besides P-bodies, plant cells have cytoplasmic foci related to RNA metabolism, siR-bodies, which are required for siRNA biogenesis [39,40], and stress granules where mRNAs are stored when translation initiation in the cell is inhibited [41,42]. It will be interesting to test whether AtRRP44B/SOV foci merge with these known cytoplasmic foci. 

Previously, Zhang et al. showed that AtRRP44A is required for female gametophyte development. Our genetic analysis confirmed their results (Tables 1, S1, and S2). In addition, the defect of AtRRP44A did not affect male gametophytes. This phenotype is similar to that of AtRRP41 [10], suggesting that AtRRP44A and AtRRP41 work together and target the same RNAs in female gametophytes. In contrast, deletion or down regulation of AtRRP44B/SOV did not affect plant viability under normal growth conditions (Figure 6B). In *S. pombe*, DIS3L2, the closest homolog of AtRRP44B/SOV is suggested to work cooperatively with the 5ʹ–3ʹ exoribonuclease XRN1 or LSM1, a subunit of the LSM1–7 complex, which activates decapping of RNAs [43]. The *S. pombe dis3l2* single mutant did not have much impact on cell growth, but the *dis3l2 xrn1Δ* double mutant showed the synthetically lethal phenotype [36]. In addition, the *dis3l2 lsm1Δ* double mutant showed a slower growth phenotype, suggesting DIS3L2 works with at least two RNA degradation pathways, LSM1 and XRN1. These two genes are conserved in *Arabidopsis* as AtLSM1 [44,45] and AtXRN4 [42], [46] both of which localize to P-bodies. It will be interesting to test whether plant AtRRP44B/SOV works with AtLSM1 and/or AtXRN4 by crossing with available T-DNA insertion mutants.

Based on our results and the available data [10,14,27], we present a model to illustrate the roles of *Arabidopsis* AtRRP44A and AtRRP44B/SOV in RNA processing and/or degradation (Figure 7). In the nucleus, AtRRP44A and the exosome core process functional noncoding RNAs like rRNA intermediates, snoRNAs and MRP RNAs. In the cytoplasm, AtRRP44B/SOV is localized to cytoplasmic foci and degrades subsets of mRNAs, the AT5G11090 mRNA for example, playing a role independent of the exosome core.

During this study we established tissue-specific knock-down mutants of *AtRRP44A* using artificial microRNA driven by mesophyll-specific *CAB3* promoter. By applying this approach, we successfully knocked down *AtRRP44A*. Different from chemical-inducible knock-down, tissue specific knock-down of essential genes enabled us to obtain viable mutants and avoid lethal phenotypes (Figure 2). Our data suggest that the tissue specific knock-down method is a useful tool to investigate essential genes that are necessary for viability. 

## Materials and Methods

### Plant materials and growth conditions

The *Arabidopsis* T-DNA insertion lines *rrp44a-1* (SALK_037533), *rrp44a-2* (SALK_141741), *rrp44a-3* (SALK_051800), *rrp44b-1* (SAIL_804_F05), *rrp44b-2* (SALK_017934) and *rrp44b-3* (SALK_010765) were selected by PCR-based genotyping using the following sets of primers: *rrp44a-1* (SALK_037533_LP, SALK_037533_RP and LBa1), *rrp44a-2* (SALK_141741_LP, SALK_141741_RP and LBa1), *rrp44a-3* (SALK_051800(2)-LP, SALK_051800(2)-RP and LBa1), *rrp44b-1* (SAIL_804_F05-LP, SAIL_804_F05-RP and SAIL_LB3), *rrp44b-2* (SALK_017934-LP, SALK_017934-RP and LBa1) and *rrp44b-3* (SALK_010765-LP, SALK_010765-RP and LBb1.3). See Table S3 for primer sequences. Plants were grown on an agar-solidified half-strength Murashige and Skoog (MS) medium or on mixed Jiffy mix (Sakata Seed Corp., Yokohama, Japan) and vermiculite soil at 23°C under continuous light.

### Plasmids

Plasmids used in this study are listed in Table S4. See Methods S1 for details of the plasmids.

### *S. cerevisiae* complementation experiment

*S. cerevisiae* strain BSY1883 [*KanMX6: TetOFF-DIS3*] and the plasmid pBS3269: [pFL36]*lys2∆: DIS3wt-TEV-PA* (for expression of ScRrp44/Dis3 in *S. cerevisiae*) were kindly provided by Bertrand Séraphin [21]. For expression of AtRRP44A, AtRRP44B/SOV, AtRRP44A plus AtRRP44B/SOV, ScRR44 and empty vectors, pOH016 plus p414-ADH, p415-ADH plus pOH022, pOH016 plus pOH022, pBS3269 plus p414-ADH and p415-ADH plus p414-ADH were introduced into the BSY1883 strain, respectively (Figure 1B). For expression of AtRRP44AHA, ScRrp44 and an empty vector, pOH001.3, pBS3269 and p415-ADH were introduced into the BSY1883 strain, respectively (Figure S1). Cultures in synthetic complete medium without leucine (LEU) and tryptophan (TRP) (Figure 1B) or without LEU (Figure S1) were grown at 30°C. In the BSY1883 strain, the chromosomal ScRrp44/Dis3 can be repressed by the addition of doxycycline (20 µg mL^−1^). Ten-fold dilution series of each transformant were spotted on LEU and TRP minus (Figure 1B) or LEU minus (Figure S1) plates with doxycycline (endogenous ScRrp44 was repressed) or without (endogenous ScRrp44 was expressed). Following selection at 30°C, growth was monitored for 90 hours at 30°C (Figure 1B) or at 17, 22, 23, 30, 34 and 37°C (Figure S1). The expression of the AtRRP44A-HA fusion protein was confirmed by western blotting using anti-HA High Affinity (Rat monoclonal antibody [clone 3F10]; Roche, Basel, Switzerland), horseradish peroxidase (HRP) conjugated anti-Rat IgG antibody and Luminata Forte Western HRP Substrate (MILLIPORE, Billerica, MA, USA).

### Northern blot analysis

For northern blot analysis, 5 µg of total RNA was separated on 7 M urea-6% polyacrylamide gels in TBE buffer (45 mM Tris, 45 mM Boric acid, 1 mM EDTA pH 8.0). RNA was electroblotted onto Hybond-N^+^ membranes (GE Healthcare, Little Chalfont, UK). The DNA oligonucleotide probes specific for rRNAs were end-labeled with γ-32P-ATP using T4 polynucleotide kinase (Toyobo, Osaka, Japan). Hybridization was performed at 42°C using PerfectHyb Plus Hybridization buffer (Sigma-Aldrich, St. Louis, MO, USA). See Table S3 for oligonucleotide probe sequences.

### Quantitative Real Time PCR (qRT-PCR)

For quantification of AtRRP44A mRNAs in the WT and the *rrp44aKD-1* and *rrp44aKD-2* mutants, three rosette leaves of 20 to 30 plants at 25 days after germination were pooled for RNA extraction. Total RNA for qRT-PCR analysis was extracted using RNAiso Plus reagent (TaKaRa, Kusatsu, Japan) according to the manufacture’s protocol. cDNA was synthesized using a PrimeScript RT reagent Kit with gDNA Eraser (TaKaRa). Any genomic DNA in the total RNA was eliminated with gDNA Eraser. The RNA was then reverse transcribed by PrimeScript RT with random hexamer primers according to the manufacturer’s instructions. Following the reaction, the cDNA was diluted 1/5. All qPCR reactions were performed as previously reported [47]. The primers used are listed in Table S3. All qRT-PCR experiments were performed under Minimal Information for Publication of Quantitative Real-Time PCR Experiments (MIQE) guidelines [48].

### Virus

pTCV-t1d1, a plasmid containing full-length cDNA of TCV downstream from T7 RNA polymerase promoters, was kindly provided by Dr. Jack T. Morris. Virions were prepared as described previously [49]. Virions were diluted at 50 µg/mL with 0.05M sodium phosphate (pH7.0) for inoculation.

## Supporting Information

Figure S1 **AtRRP44AHA does not complement the S. *cerevisiaerrp44* doxycycline (DOX) repressible mutant.** (A) Growth phenotypes resulting from expression of plasmid-borne AtRRP44A-HA, *S. cerevisiae* Rrp44 (ScRrp44) and negative control alleles were assessed in the presence (repressed chromosomal ScRrp44) or absence (expressed chromosomal ScRrp44) of DOX after incubation for 120 h at 17, 22, 23, 30, 34 or 37°C. –LEU, without leucine. (B) AtRRP44AHA expression was determined by western blot analysis in the S. *cerevisiae* repressible *rrp44* mutant without DOX. AtRRP44AHA protein was extracted from yeast cultured at 30 °C.
(TIF)

Figure S2 **Activity of the CAB3 promoter in leaf tissue.** *Arabidopsis* CAB3 promoter activity was determined by staining of *Arabidopsis* transformants expressing the GUS reporter gene under the control of about 1.5 kb genomic sequence containing the CAB3 promoter. 20 days post-germination (dpg) 5th rosette leaf (Left panel) and a transverse section of the leaf (Right panel).
(TIF)

Figure S3 **Levels of mRNAs in knocked-down mutants.** The amounts of AtRRP4 mRNA in *gusKD-2#2-10-3*, *rrp4KD-2*#*2-6* and *rrp4KD-3*#*4-2*, and the amounts of AtRRP41 mRNA in *gusKD*-2#2-10-3, *rrp41KD-1*#*3-3* and *rrp41KD-3#9-2* were analyzed by qRT-PCR. Total RNAs were isolated from 25 dpg rosette leaves. Error bars represent standard errors. Six biological replicates and two technical replicates were performed. * indicates significant difference (*p* < 0.01, Tukey’s test).
(TIF)

Figure S4 **TCV genomic RNA accumulation was not affected by AtRRP41, AtRRP44A or AtRRP44B/SOV.** (A) TCV genomic RNA structure. qRT-PCR of TCV accumulation in *rrp41KD-1#3* and *#9* (B), in *rrp44aKD-1* and *-2* (C), and in *rrp44b-1* and *-2* (D). TCV virions were inoculated at 16 dpg onto 4th and 5th rosette leaves, which were then collected at 3 days post-inoculation (dpi) for total RNA isolation. Error bars represent standard errors. Six biological replicates and two technical replicates were performed. Each biological replicate included six plants. EF1a mRNA was used as an endogenous control.
(TIF)

Table S1 ***rrp44a* transmission in the selfed progeny of heterozygous *rrp44a*/+ mutants.**
(XLSX)

Table S2 **Germination of the selfed progeny of heterozygous *rrp44a*/+ mutants.**
(XLSX)

Table S3 **Oligonucleotides used in this study.**
(XLSX)

Table S4 **Plasmids used in this work.**
(XLSX)

Method S1 **Plasmid construction.**
(DOCX)
