# Supplementary figures and images for: Arabidopsis AtRRP44A Is the Functional Homolog of Rrp44/Dis3, an Exosome Component, Is Essential for Viability and Is Required for RNA Processing and Degradation
Source: PLoS One. 2013 Nov 7;8(11):e79219. doi: 10.1371/journal.pone.0079219 (PMC3820695; doi:10.1371/journal.pone.0079219)

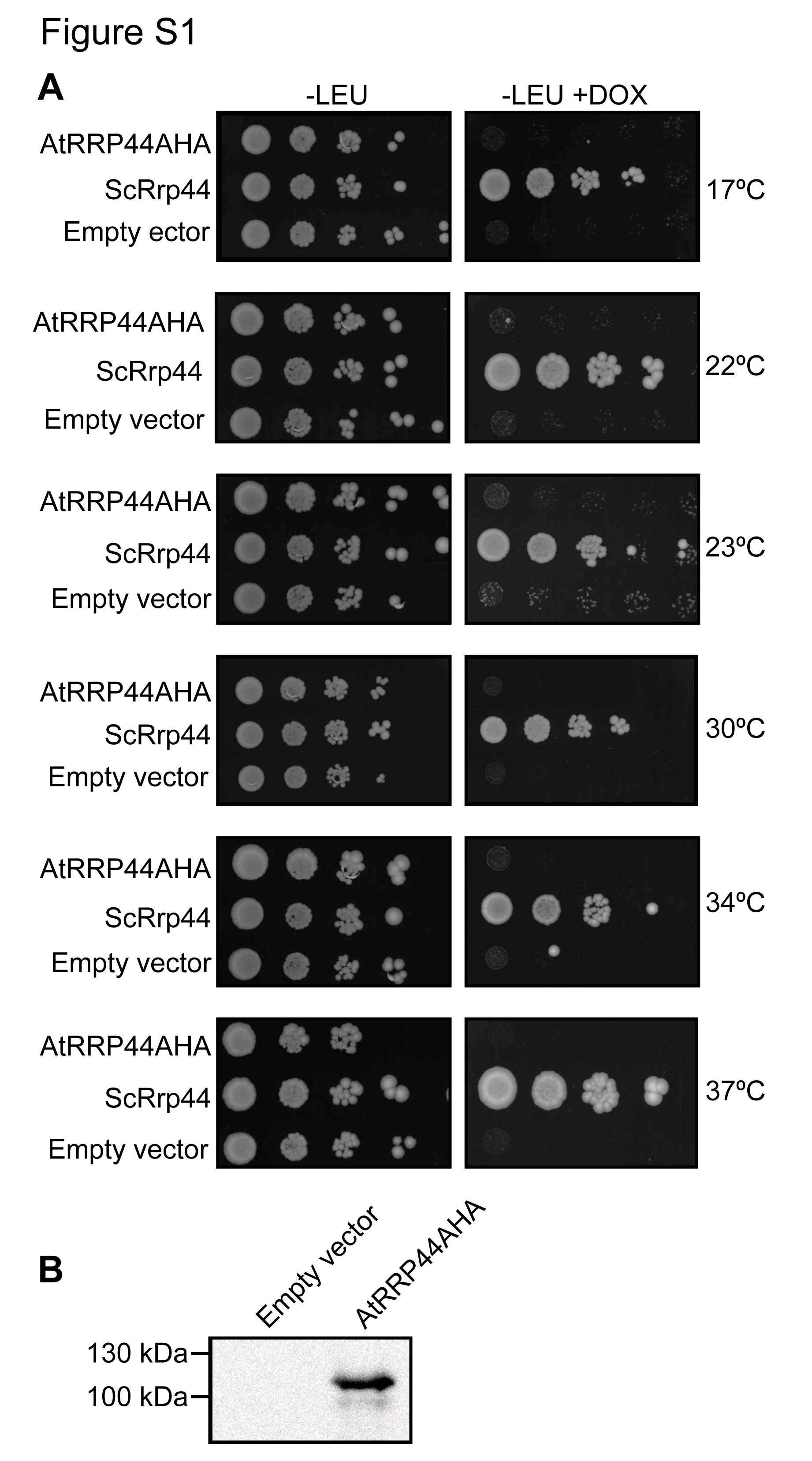

Supplement: Figure S1 — AtRRP44AHA does not complement the S. cerevisiaerrp44 doxycycline (DOX) repressible mutant. (A) Growth phenotypes resulting from expression of plasmid-borne AtRRP44A-HA, S. cerevisiae Rrp44 (ScRrp44) and negative control alleles were assessed in the presence (repressed chromosomal ScRrp44) or absence (expressed chromosomal ScRrp44) of DOX after incubation for 120 h at 17, 22, 23, 30, 34 or 37°C. –LEU, without leucine. (B) AtRRP44AHA expression was determined by western blot analysis in the S. cerevisiae repressible rrp44 mutant without DOX. AtRRP44AHA protein was extracted from yeast cultured at 30 °C. (TIF) [file pone.0079219.s001.tif]

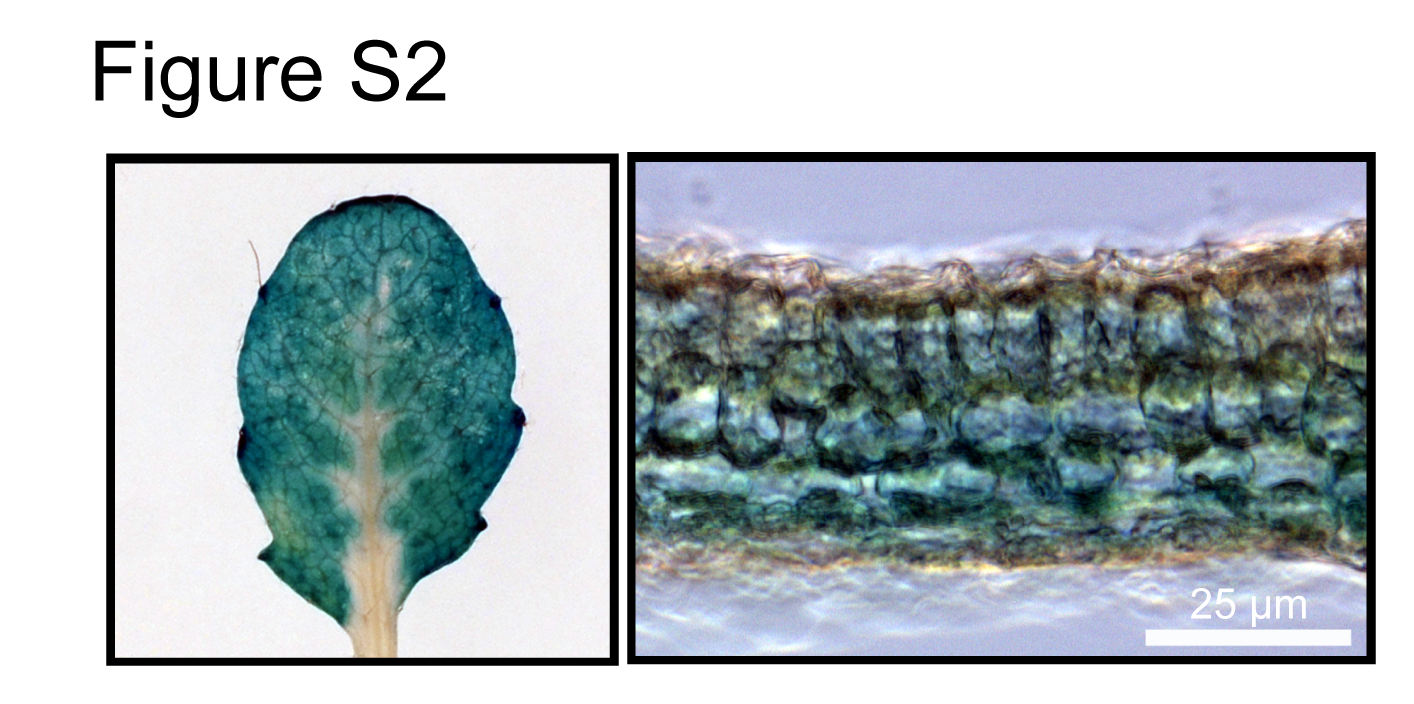

Supplement: Figure S2 — Activity of the CAB3 promoter in leaf tissue. Arabidopsis CAB3 promoter activity was determined by staining of Arabidopsis transformants expressing the GUS reporter gene under the control of about 1.5 kb genomic sequence containing the CAB3 promoter. 20 days post-germination (dpg) 5th rosette leaf (Left panel) and a transverse section of the leaf (Right panel). (TIF) [file pone.0079219.s002.tif]

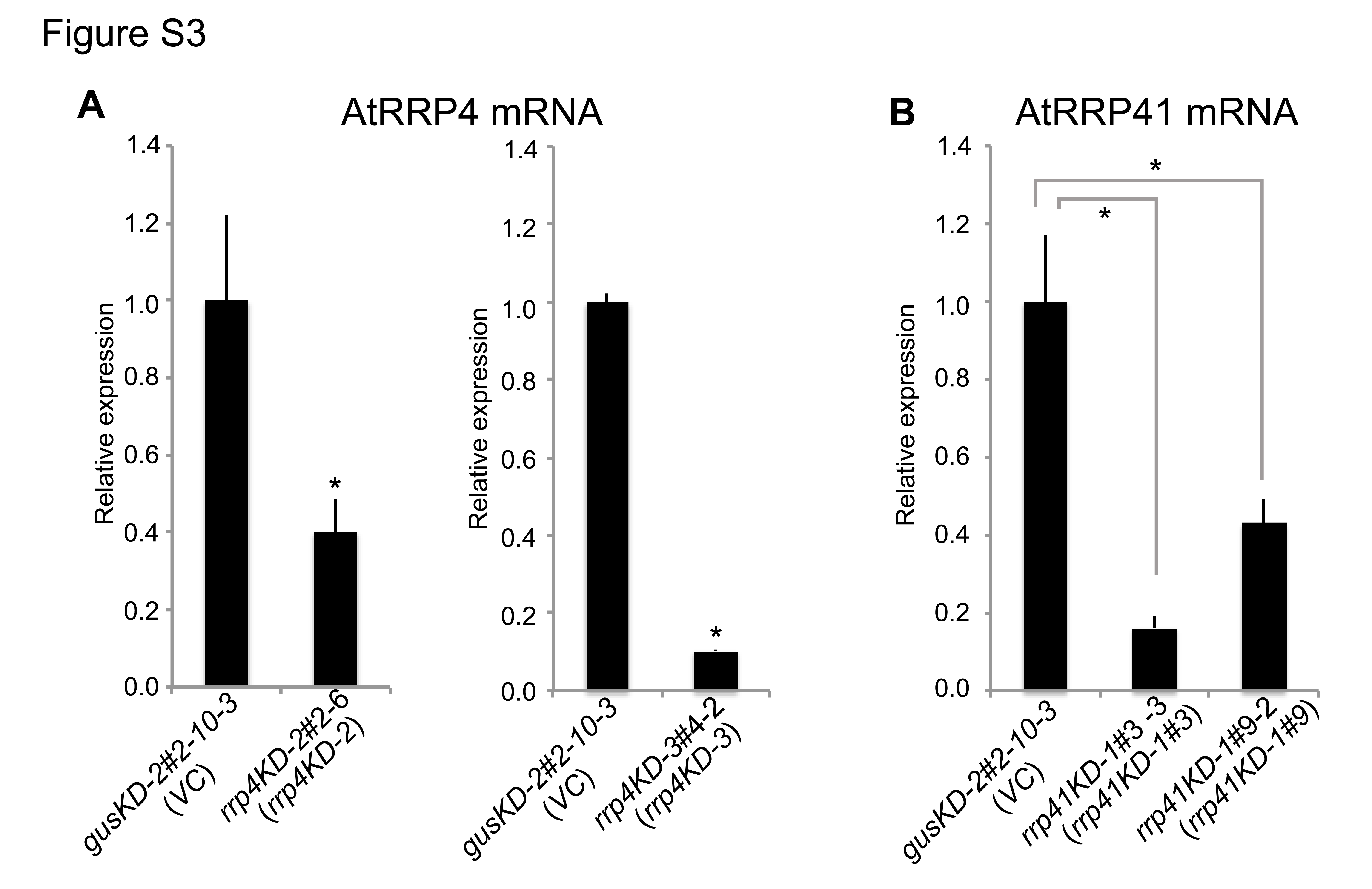

Supplement: Figure S3 — Levels of mRNAs in knocked-down mutants. The amounts of AtRRP4 mRNA in gusKD-2#2-10-3, rrp4KD-2#2-6 and rrp4KD-3#4-2, and the amounts of AtRRP41 mRNA in gusKD-2#2-10-3, rrp41KD-1#3-3 and rrp41KD-3#9-2 were analyzed by qRT-PCR. Total RNAs were isolated from 25 dpg rosette leaves. Error bars represent standard errors. Six biological replicates and two technical replicates were performed. * indicates significant difference (p < 0.01, Tukey’s test). (TIF) [file pone.0079219.s003.tif]

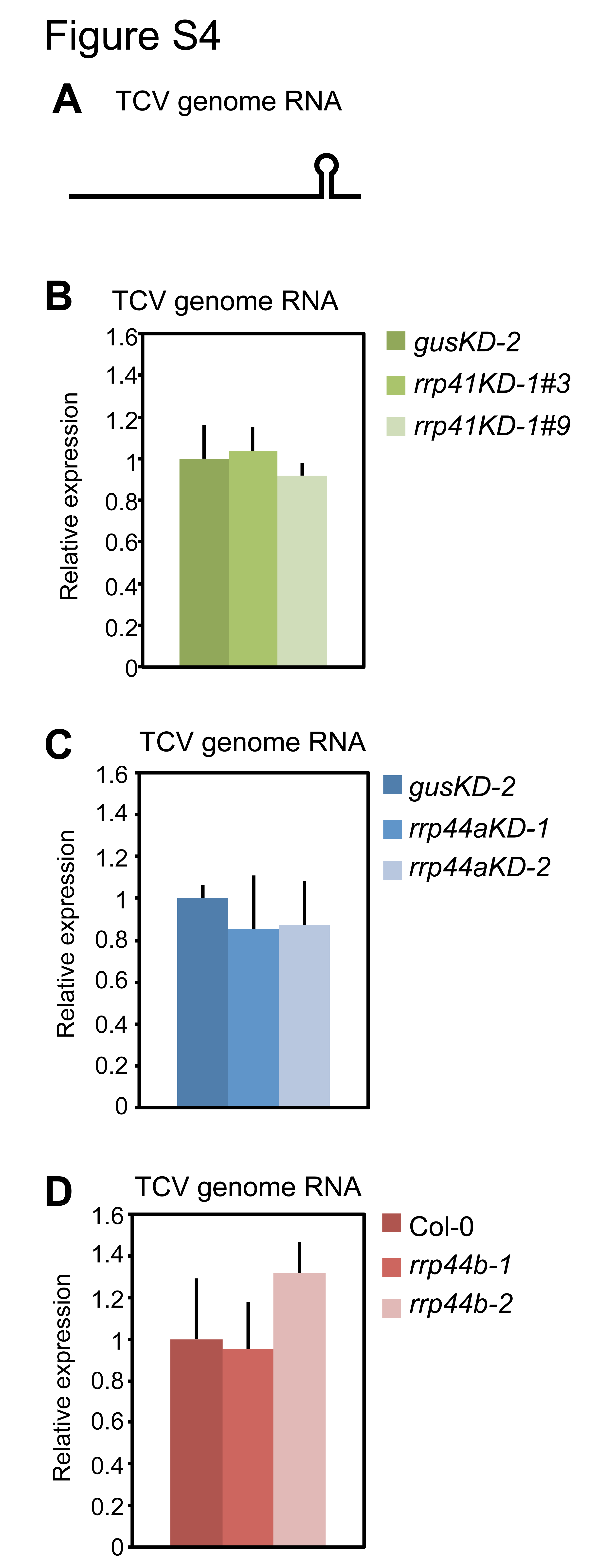

Supplement: Figure S4 — TCV genomic RNA accumulation was not affected by AtRRP41, AtRRP44A or AtRRP44B/SOV. (A) TCV genomic RNA structure. qRT-PCR of TCV accumulation in rrp41KD-1#3 and #9 (B), in rrp44aKD-1 and -2 (C), and in rrp44b-1 and -2 (D). TCV virions were inoculated at 16 dpg onto 4th and 5th rosette leaves, which were then collected at 3 days post-inoculation (dpi) for total RNA isolation. Error bars represent standard errors. Six biological replicates and two technical replicates were performed. Each biological replicate included six plants. EF1a mRNA was used as an endogenous control. (TIF) [file pone.0079219.s004.tif]
