# Supplementary material for: Arabidopsis AtRRP44A Is the Functional Homolog of Rrp44/Dis3, an Exosome Component, Is Essential for Viability and Is Required for RNA Processing and Degradation
Source: PLoS One. 2013 Nov 7;8(11):e79219. doi: 10.1371/journal.pone.0079219 (PMC3820695; doi:10.1371/journal.pone.0079219)
Supplement: Method S1 — Plasmid construction. (DOCX) [file pone.0079219.s009.docx]

**Supplementary methods**

**pWAT000**

A binary vector, pGreenII0000 was obtained from John Innes Centre [1] (http://www.pgreen.ac.uk/). A PCR fragment was amplified from pGreenII0000 using the primers pGN-MCS-F and Kan-dBsmBI-R, and another was amplified from pGreenII0000 using the primers Kan-dBsmBI-F and pGN-Kan-R. We then amplified a PCR fragment from a mixture of these two PCR fragments using the primers pGN-MCS-F and pGN-Kan-R, and the *Hin*dIII–*Nru*I fragment of the amplified DNA was substituted for the small *Hin*dIII–*Nru*I fragment of pGreenII0000 to construct pWAT000.

**pWAT2**

pWAT2 is a pWAT000 derivative containing a glufosinate ammonium resistance gene under controls of an ocs promoter and an ocs terminator. The CD3-1057 (pPZP-RCS2-bar) plasmid was obtained from Arabidopsis Biological Resource Center. A PCR fragment (PCR-1) was amplified from pPZP-RCS2-bar using the primers Hpa-ocsP-F and ocsP(-)BsaI-R, a PCR fragment (PCR-2) was amplified from pPZP-RCS2-bar using the primers ocsP(-)BsaI-F and ocsP-bar-R, a PCR fragment (PCR-3) was amplified from 35S-bar, which had been obtained from John Innes Centre, using the primers ocsP-bar-F and ocsT-bar-R, and a PCR fragment (PCR-4) was amplified from pPZP-RCS2-bar using the primers bar-ocsT-F and Hpa-ocsT-R. We then amplified a PCR fragment (PCR-5) from a mixture of the PCR-1 and the PCR-2 fragments using the primers Hpa-ocsP-F and ocsP-bar-R, and amplified a PCR fragment (PCR-6) from a mixture of the PCR-3 and the PCR-4 fragments using the primers ocsP-bar-F and Hpa-ocsT-R. We then amplified a PCR fragment (PCR-7) from a mixture of the PCR-5 and the PCR-6 fragments using the primers Hpa-ocsP-F and Hpa-ocsT-R. Then, three PCR fragments named PCR-8, PCR-9, and PCR-10 were amplified from the PCR-7 fragment using the primer sets Hpa-ocsP-F plus bar-dBsmBI-R, bar-dBsmBI-F plus ocsT-dBsmBI-R, and ocsT-dBsmBI-F plus Hpa-ocsT-R, respectively. We then amplified a PCR fragment (PCR-11) from a mixture of the PCR-8, the PCR-9 and the PCR-10 fragments using the primers Hpa-ocsP-F and Hpa-ocsT-R. Then, the *Hpa*I fragment of the PCR-11 was inserted into the *Hpa*I site on pWAT000 to construct pWAT2-PS. To further remove restriction enzyme sites, three PCR fragments named PCR-12, PCR-13, and PCR-14 were amplified from pWAT2-PS using the primer sets pGN-LB-F plus Bar-dSacII-F, Bar-dSacII-R plus Bar-dSaldBsmB-F, and Bar-dSaldBsmB-R plus pGN-Hpa-R, respectively. We then amplified a PCR fragment (PCR-15) from a mixture of the PCR-13 and the PCR-14 fragments using the primers Bar-dSacII-R and pGN-Hpa-R. We then amplified a PCR fragment (PCR-16) from a mixture of the PCR-12 and the PCR-15 fragments using the primers pGN-LB-F and pGN-Hpa-R, and the *Hpa*I fragment of the PCR-16 was inserted into the *Hpa*I site in pWAT2-PS to construct pWAT2. The orientation of the insert in pWAT2-PS and pWAT2 was selected with PCR using the primers ocsT-410R and pGN-Hpa-R.

**pWAT208**

pWAT208 is a pWAT2 derivative containing a mesophyll-specific CAB3 promoter and an *Agrobacterium* 6b terminator. To remove a set of restriction enzyme sites in the CAB3 promoter, we amplified a PCR fragment (PCR-17) from *Arabidopsis* Col-0 genome DNA using the primers CAB3Pro-F and CAB3Pro-R. Then, five PCR fragments named PCR-18, PCR-19, PCR-20, PCR-21, and PCR-22 were amplified from the PCR-17 fragment using the primer sets Kpn-CAB3Pro-F plus CAB3P-24R, CAB3P-26F plus CAB3P-55R, CAB3P-57F plus CAB3P-58R, CAB3P-60F plus CAB3P-129R, and CAB3P-131F plus Xho-CAB3Pro-R, respectively. We then amplified a PCR fragment (PCR-23) from a mixture of the PCR-18 and the PCR-19 fragments using the primers Kpn-CAB3Pro-F and CAB3P-55R. We then amplified a PCR fragment (PCR-24) from a mixture of the PCR-20 and the PCR-21 fragments using the primers CAB3P-57F and CAB3P-129R. Then, a PCR fragment (PCR-25) was amplified from a mixture of the PCR-22 and the PCR-25 fragments using the primers CAB3P-57F and Xho-CAB3-Pro-R. We then amplified a PCR fragment (PCR-26) from a mixture of the PCR-23 and the PCR-26 fragments using the primers Kpn-CAB3Pro-F and Xho-CAB3Pro-R. Then, the *Kpn*I–*Xho*I fragment of the PCR-26 fragment was substituted for the small *Kpn*I–*Xho*I fragment of pWAT2 to construct pWAT208-PS.

A PCR fragment (PCR-27) was amplified using the primers 6b-Ter-F1 and 6b-Ter-R1. We then amplified a PCR fragment (PCR-28) from the PCR-27 fragment using the primers Xba-6b-Ter-F2 and Sac-6b-Ter-R2. Then, the *Xba*I–*Sac*I fragment of the PCR-28 fragment was substituted for the small *Xba*I–*Sac*I fragment of pWAT208-PS to construct pWAT208.

**pWAT208-MASS-GUS**

A PCR fragment (PCR-29) was amplified from pENTR-gus (Invitrogen) using primers Xho-MASS-GUS-F and Xba-GUS-R. Then, the *Xho*I–*Xba*I fragment of the PCR-29 fragment was substituted for the small *Xho*I–*Xba*I fragment of pWAT208 to construct pWAT208-MASS-GUS [2].

**pWAT208-MIR390Bsa**

We amplified a PCR fragment (PCR-30) from *Arabidopsis* Col-0 genome DNA using the primers Sal-MIR390a-F and Spe-MIR390a-R. Then, two PCR fragments named PCR-31 and PCR-32 were amplified from the PCR-30 fragment using the primer sets Sal-MIR390a-F plus Bsa-390R and Bsa-390F plus Spe-MIR390a-R, respectively. We then amplified a PCR fragment (PCR-33) from a mixture of the PCR-31 and the PCR-32 fragments using the primers Sal-MIR390a-F and Spe-MIR390a-R. Then, the *Sal*I–*Spe*I fragment of the PCR-33 fragment was substituted for the small *Sal*I–*Spe*I fragment of pWAT208 to construct pWAT208-MIR390Bsa.

**pSoup-Spec**

A helper plasmid pSoup, which is required for the replication of the pGreenII0000 derivatives in *Agrobacterium*, was obtained from John Innes Centre [1] (http://www.pgreen.ac.uk/). Because an *A. tumefaciens* C58 strain frequently becomes resistant to the tetracycline [3], we replaced the tetracycline resistance gene on pSoup with a spectinomycin resistance gene as follows; two PCR fragments named PCR-34 and PCR-35 were amplified from pSoup using the primer sets HindIII-pSoup-F plus SpecR-Pro-R and SpecR-Ter-F plus NdeI-pSoup-R, respectively. A PCR fragment PCR-36 was amplified from pPZP-RCS2-bar using the primers pSoup-SpecR-F and pSoup-SpecR-R. We then amplified a PCR fragment (PCR-37) from a mixture of the PCR-34 and the PCR-36 fragments using the primers HindIII-pSoup-F and pSoup-SpecR-R. We then insert the PCR-37 fragment into pCR4zerobluntTOPO vector (Invitrogen) to construct pCR4-Spec. Then, the *Hin*dIII–*Nde*I fragment of pCR4-Spec was substituted for the small *Hin*dIII–*Nde*I fragment of pSoup to construct pSoup-Spec.

**pNK009 to pNK015**

PCR fragments were amplified by using primers listed below (PCR-39: 390amiR_RRP4_2-F and 390amiR_RRP4_2-R, PCR-40: 390amiR_RRP4_3-F and 390amiR_RRP4_3-R, PCR-41: 390amiR_RRP41_1-F and 390amiR_RRP41_1-R, PCR-42: 390amiR_RRP41_3-F and 390amiR_RRP41-3-R, PCR-43: 390amiR_RRP44A_1-F and 390amiR_RRP44A_1-R, PCR-44: 390amiR_RRP44A_2-F and 390amiR_RRP44A_2-R and PCR-45: 390amiRgus-2-F and 390amiRgus-2-R). Then, the *Bsa*I fragments of PCR-39 to PCR-45 were substituted for the small *Bsa*I fragment of pWAT208-MIR390Bsa to construct pNK009, pNK010, pNK011, pNK012, pNK013, pNK014 and pNK015, respectively.

**pOH016**

A PCR fragment (PCR-46) was amplified from *Arabidopsis* Col-0 cDNA using primers F_44A-CDS_pOH016 and R_44A-CDS_pOH016. Then, PCR-39 and the large *Sma*I*-Xho*I fragment of p415-ADH [4] were assembled with In-Fusion HD Cloning Kit (Takara) to construct pOH016.

**pOH022**

A PCR fragment (PCR-47) was amplified from *Arabidopsis* Col-0 cDNA using primers F_SmaI_44BCDS and R_44BCDS_pOH022. Then, the *Sma*I-*Sal*I fragment of PCR-47 was substituted for the small *Sma*I-*Sal*I fragment of p414-ADH [4] to construct pOH022.

**pOH001.3**

A PCR fragment (PCR-48) was amplified from *Arabidopsis* Col-0 cDNA using the primers F_p415_cAt44A and R_p415_cAt44A. Then, a PCR fragment (PCR-49) was amplified using F_RRP44A_3HA and R_3HA_RRP44A_XhoI. We then amplified a PCR fragment (PCR-50) from a mixture of the PCR-48 and the PCR-49 fragments using the primers F_p415_cAt44A and R_3HA_RRP44A_XhoI. The *Sma*I-*Sal*I fragment of PCR-50 was substituted for the small *Sma*I-*Sal*I fragment of p415-ADH to construct pOH001.3.

1. Hellens RP, Edwards EA, Leyland NR, Bean S, Mullineaux PM (2000) pGreen: a versatile and flexible binary Ti vector for Agrobacterium-mediated plant transformation. Plant Mol Biol 42: 819–832.

2. Sawant SV, Kiran K, Singh PK, Tuli R (2001) Sequence architecture downstream of the initiator codon enhances gene expression and protein stability in plants. Plant Physiol 126: 1630–1636.

3. Luo ZQ, Farrand SK (1999) Cloning and characterization of a tetracycline resistance determinant present in Agrobacterium tumefaciens C58. J Bacteriol 181: 618–626.

4. Mumberg D, Müller R, Funk M (1995) Yeast vectors for the controlled expression of heterologous proteins in different genetic backgrounds. Gene 156: 119–122. Available: http://eutils.ncbi.nlm.nih.gov/entrez/eutils/elink.fcgi?dbfrom=pubmed&id=7737504&retmode=ref&cmd=prlinks.
